# Usability Evaluation of a Virtual Reality Multisensory Sham-Feeding Device for Patients Undergoing Fasting Periods for Colorectal Cancer Surgery: Mixed Methods Study

**DOI:** 10.2196/75641

**Published:** 2025-10-08

**Authors:** Xingzhu Yuan, Peiling Ye, Xinyue Liu, Yuehan Hu, Qin Xu, Wenyi Zhao, Ka Li

**Affiliations:** 1 West China School of Nursing/Department of Medicine and Engineering Interdisciplinary Research Laboratory of Nursing & Materials West China Hospital Sichuan University Chengdu China; 2 Division of Gastrointestinal Surgery, Department of General Surgery West China Hospital Sichuan University Chengdu China; 3 Department of Obstetric Nursing West China Second University Hospital Sichuan University Chengdu China; 4 Department of Medicine and Engineering Interdisciplinary Research Laboratory of Nursing & Materials West China Hospital Sichuan University Chengdu China

**Keywords:** colorectal cancer, fasting, virtual reality, multisensory stimulation, patient comfort, appetite, System Usability Scale, mixed methods research

## Abstract

**Background:**

Colorectal cancer surgery requires perioperative fasting to ensure safety, but this can cause physiological and psychological discomfort, such as impaired intestinal motility, bloating, immune suppression, anxiety, and appetite loss. To address this challenge, we developed the virtual reality (VR) multisensory sham-feeding device (VRMS-SFD), based on Pavlov conditioned reflex mechanism, where vagal stimulation triggered by seeing, smelling, or thinking about food activates cephalic-phase responses, promoting digestive secretion and intestinal motility. The device integrates multisensory stimulation—visual (food presentation), auditory (eating sounds and relaxing music), and olfactory (food-specific scents)—to create a VR dining experience. It features three VR scenes (Chinese restaurant, fruit shop, and dessert shop) with 23 food options, offering immersive interaction through a head-mounted display and synchronized scent release.

**Objective:**

This study aimed to evaluate the usability, acceptability, and safety of the VRMS-SFD in patients with colorectal cancer during postoperative fasting, assessing its potential to alleviate discomfort, stimulate appetite, and enhance emotional well-being.

**Methods:**

A mixed methods design was used. Participants used VRMS-SFD for 20 minutes during each meal time over 3 postoperative days. Quantitative data included the System Usability Scale (SUS) and demographic or clinical variables. Qualitative data were obtained from 15- to 30-minute semistructured interviews, transcribed verbatim, and thematically analyzed using NVivo (version 15.0; QSR International). When no new themes emerged, the sample size was considered sufficient, and data saturation was achieved. Two researchers independently coded transcripts, with discrepancies resolved by a third reviewer. The study was ethically approved (1087) and clinically registered.

**Results:**

A total of 37 patients were included. The mean SUS score was 77.78 (SD 7.90; range 62.5-97.5), indicating high usability. Participants rated ease of use (mean 4.46, SD 0.56), learnability (mean 4.27, SD 0.69), and confidence (mean 4.27, SD 0.61) positively. There was no correlation between SUS score and age (*r*=0.05). Thematic analysis revealed four themes: (1) immersive and enjoyable experience, with patients describing the device as “novel” and “engaging”; (2) reduced fasting-related discomfort, including less bloating, improved mood, and reported peristalsis; (3) appetite stimulation, with many noting increased hunger; and (4) improvement suggestions, such as enhancing scent authenticity, simplifying controls, and diversifying music. One participant experienced transient dizziness, resolving within 5 minutes post–device removal, with no other adverse events. Quantitative and qualitative findings converged, confirming robust usability and clinical benefits.

**Conclusions:**

The VRMS-SFD may be a feasible, well-accepted tool that mitigates fasting-related discomfort, enhances appetite, and improves emotional well-being in patients undergoing colorectal cancer surgery. Future iterations should refine scent calibration and the user interface. Larger trials with objective measures (eg, gastrointestinal motility and hormone levels) are needed to validate efficacy and explore applications in other conditions.

**Trial Registration:**

Chinese Clinical Trial Registry (ChiCTR), ChiCTR2100051419; https://www.chictr.org.cn/showprojEN.html?proj=134263

## Introduction

### Background

Colorectal cancer is the third most common malignancy and the second leading cause of cancer-related mortality worldwide, with over 1.9 million new cases and 904,000 deaths reported in 2022 [1]. Surgery is the primary treatment for most patients with colorectal cancer [2]. To minimize perioperative risks, fasting protocols are mandatory for patients, typically requiring 6 hours of preoperative fasting and withholding oral intake until the return of bowel function postoperatively [3]. However, prolonged perioperative fasting (48-120 h) may negatively impact the intestinal mechanical and immune barriers, leading to intestinal ischemia, reperfusion injury, and postoperative gastrointestinal dysfunction [4,5]. Postoperative ileus occurs in approximately 10%-27% of patients following major abdominal surgery, resulting in abdominal distension, delayed recovery, and increased hospital stays [6,7]. Strategies such as early enteral nutrition, early ambulation, and probiotics are incorporated in enhanced recovery protocols to promote gut function, but each has notable limitations, including aspiration risk and inconsistent efficacy [8-10].

Sham feeding, a technique based on Pavlov conditioned reflex theory, involves multisensory stimulation without actual ingestion, and has been shown to stimulate gastric acid secretion, enhance intestinal motility, and support gut function [11]. Based on this, Li et al [12] proposes a new concept of “sham feeding”: simulating oral food intake by integrating visual, auditory, and olfactory sensory stimuli to induce cephalic phase secretion in brain neurons, thereby addressing the shortcomings of traditional nutritional infusion, which lacks the sensory stimulation of food’s color, aroma, and taste, making it difficult to trigger cephalic phase secretion. Simple forms such as gum chewing have demonstrated benefits in patients with colorectal cancer [13,14]. Virtual reality (VR) technology provides immersive, digital environments capable of delivering visual, auditory, and olfactory stimuli simultaneously [15,16]. While VR has been applied in clinical settings for anxiety reduction, pain management, and rehabilitation [17,18]. Meanwhile, VR interventions have shown potential in managing appetite-related behaviors. Recent studies have started to explore the effects of VR on appetite and eating behaviors. Huang et al [19] found that immersive VR reminiscence interventions led to sustained improvements in neuropsychiatric symptoms and appetite in people with dementia. Similarly, Sauchelli and Brunstrom [20] reported that VR exergaming enhanced the affective experience of exercise and significantly reduced postexercise energy intake by 12% in inactive adults. These findings suggest that VR-based sensory stimulation may influence food-related cognition and appetite regulation. However, systematic reviews such as Xia et al [21] indicate that evidence regarding VR’s direct effect on appetite remains inconclusive. However, its use for gut function regulation during perioperative fasting remains underexplored, highlighting the need for further targeted research in specific clinical contexts such as perioperative fasting.

To address this gap, we developed an immersive VR multisensory sham-feeding device (VRMS-SFD), which is designed to simulate cephalic phase responses through synchronized visual, auditory, and olfactory stimulation of food in a 360° immersive environment, thereby bridging the gap between traditional sham feeding and modern digital therapeutics.

### Study Aims and Objectives

This study aimed to evaluate the usability and user experience of a newly developed VRMS-SFD for patients with colorectal cancer during perioperative fasting. The objectives were to: (1) assess the usability of VRMS-SFD using the System Usability Scale (SUS), (2) explore patients’ subjective experiences with VRMS-SFD through semistructured qualitative interviews, (3) identify perceived benefits and barriers regarding the use of VR-based sham feeding for appetite stimulation and discomfort relief, and (4) gather user feedback to inform future iterations of the device and guide clinical implementation.

## Methods

### Study Design

The study used a mixed methods approach and used the IDEAS (Integrate, Design, Assess, and Share) framework for creating digital health behavior change interventions [22]. The IDEAS framework recommends that large-scale randomized controlled trials be preceded by smaller-scale assessments to examine preliminary efficacy and usability. In these early evaluations, usability and satisfaction can be assessed through questionnaires, while interviews provide deeper insights into user experiences [23]. In this study, qualitative and quantitative evaluation of device usability and safety for patients undergoing fasting periods for colorectal cancer surgery using our self-developed VRMS-SFD. The study has been approved by the ethics committee and clinical trial registry, and written informed consent was obtained from all participants before they joined the study. To maintain confidentiality, each participant was assigned a numerical code. No compensation was provided for participation in this study. This study followed the MMR-RHS (Mixed Methods Reporting in Rehabilitation and Health Sciences) checklist, provided in Multimedia Appendix 1 [24].

### Participants—Inclusion and Exclusion Criteria

To be eligible, participants had to (1) be inpatients in the Colorectal Cancer Center ward, (2) be aged 18 to 80 years, (3) be patients who have undergone colorectal cancer surgery, (4) be patients who can cooperate with nurses to use VR equipment, and (5) sign informed consent and voluntarily participate in the study. Exclusion criteria were the following: (1) pregnant or lactating women, or patients with psychiatric disorders; (2) patients with serious and uncontrolled organic diseases or infections, such as decompensated heart failure, lung failure, kidney failure, or other life-threatening diseases at any time; (3) patients with intestinal obstruction, gastric or intestinal perforation, chronic enteritis, or other diseases that affect intestinal function greatly; (4) patients with a history of, or at high risk of gastrointestinal ulcer, (5) patients who have used probiotics, yogurt, probiotics powder, other microecological probiotics, metformin, proton pump inhibitors, or berberine within last 1 month, (6) patients with a history of motion sickness, such as Meniere syndrome or otolithiasis; (7) patients who have undergone preoperative radiotherapy and chemotherapy; 8) patients with concomitant use of other experimental drugs; and (9) patients currently participating in other clinical trials.

### Recruitment

Potential participants are recruited through posters posted in the department or by the investigators after the patient has been admitted to the hospital. Their potential eligibility was then assessed, and if deemed eligible, they were invited to join the project for baseline data collection, intervention, and final measurement in the Colorectal Cancer Center ward of West China Hospital, Sichuan University.

### VRMS-SFD Intervention Procedures

**Figure 1:**
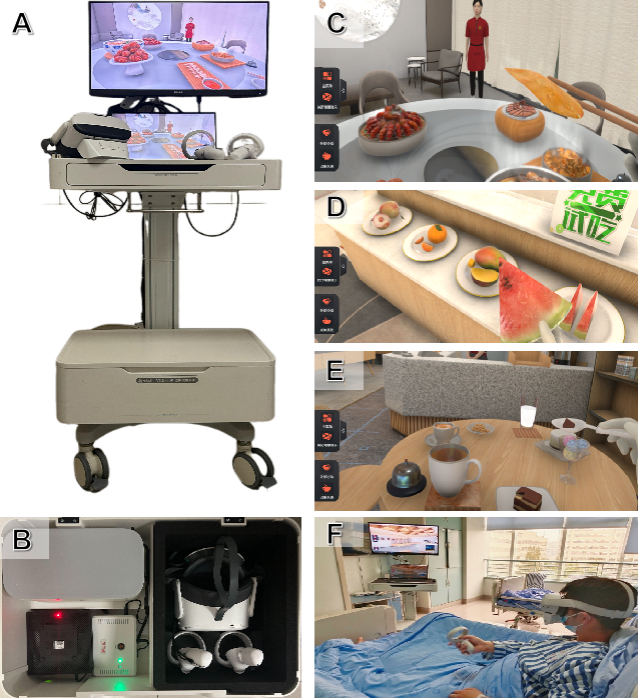
Hardware setup, immersive VR environment, and patient use of the VRMS-SFD. (A) Photograph showing the integrated hardware system, including a mobile nursing treatment cart, a high-performance laptop (running VR drivers), a 21-inch display monitor, a wireless VR headset (PICO Neo 3, with built-in audio playback), a PICO controller, and an olfactory scent-emitting box attached to the VR headset. (B) Cabinet under the cart with VR headset, controllers, and Wi-Fi 6 router. (C-E) Actual in-VR screenshots from three immersive dining scenes: a fruit shop (C), a Chinese-style restaurant (D), and a dessert shop (E). Participants can select from 23 VR food items (eg, spicy crayfish, bacon, sugar-oil glutinous balls, tiramisu, and milk). When a food item is grasped using the PICO controller and moved toward the mouth, the corresponding scent is emitted in real-time by the scent box. Each food model is rendered with frame-by-frame animation to simulate physical deformation and environmental dynamics (eg, steam and sunlight) depending on the scene. Synchronized audio, including chewing, swallowing, and ambient music, enhances the realism of the simulated eating experience. (F) A postoperative patient with colorectal cancer using the VRMS-SFD. This photograph was taken with patient consent and does not reveal identifiable facial features. It illustrates the real-world application of the device during perioperative fasting. VR: virtual reality; VRMS-SFD: virtual reality multisensory sham-feeding device.

The VRMS-SFD hardware and software were developed collaboratively by the research team at West China Hospital and an affiliated university-based engineering team. No external commercial vendors were involved. All authors contributed to the conceptualization, iterative prototyping, and pilot usability testing of the device. The complete VR system includes a wireless head-mounted display (PICO Neo 3 Pro, Pico Interactive Inc, United States), PICO controllers, an integrated aroma emitter, a custom wireless gateway, and a mobile treatment cart with a high-performance laptop and display monitor (Figure 1).

Although the PICO headset is inherently wireless, a custom-designed wireless gateway (TP-Link AX6000) was integrated to ensure real-time synchronization between the VR content and the olfactory stimuli. This gateway enables low-latency (10-20 ms) communication via a 2.4 GHz band within a 0.5-5 m range, linking the VR system and aroma emitter through a stable local area network. When users select and bring a VR food item close to the mouth using the controller, the VR apps transmit a command to the control node via the gateway. The control module translates this command into channel-specific signals, which are amplified and passed to the ultrasonic chip in the aroma emitter, instantly releasing the corresponding scent. This design ensures simultaneous delivery of visual, auditory, and olfactory stimuli, enhancing the immersive experience of VR eating. This real-time alignment, facilitated by the self-developed wireless transmitter and ultrasonic atomization technology, represents a key technical innovation, ensuring immersive multisensory coherence during VR dining.

The device features three VR dining scenes—a Chinese restaurant, a fruit shop, and a dessert shop—offering 23 VR food options such as mapo tofu, maocai, bacon, mango, orange, apple, cappuccino, ice cream, and cookies, each with matching scents. During use, patients hear detailed eating, drinking, and chewing sounds via the headset’s built-in speaker, simulating real eating steps. The environment includes light, relaxing background music to promote mental relaxation.

Patients interacted with the VRMS-SFD for 20 minutes during each meal period (7 AM-9 AM, 11 AM-1 PM, and 5 PM-7 PM) from preoperative fasting through postoperative day 3. Further hardware schematics and detailed procedural steps for equipment calibration and patient interaction are provided in Multimedia Appendix 2, and a demonstration video is available in Multimedia Appendix 3.

### Data Collection and Organization

#### Overview

The collection and collation of questionnaire data and semistructured interview data were done by two graduate nursing students who had received training. Qualitative and quantitative data were collected from participants following each session after full use of VR. The survey captured basic information about hospitalized patients. The specific content of the patient-facing questionnaire is provided in Multimedia Appendix 4.

At baseline, demographic and clinical information—including gender, age, height, weight, education level, marital status, place of residence, medical insurance coverage, and prior experience with VR—were collected using a structured questionnaire designed for this study. The data were obtained through face-to-face interviews conducted by two trained graduate nursing students before patients engaged with the VR device. Clinical data were extracted from patient charts by researchers using a standardized data collection form. Data were recorded on paper and later digitized and managed using Excel (Microsoft Corp). All responses were documented confidentially and stored securely for subsequent analysis.

#### Quantitative Measurement

Patients were required to provide their subjective assessment of their experience, in which the SUS was adopted to examine the usability and learnability of the device; it has a high reliability coefficient of 0.85 [25]. The SUS consists of ten 5-point Likert scale questions, from 1=strongly disagree to 5=strongly agree. Both positively and negatively framed items are included. For positively framed items, the corresponding score of each item should be –1; for the negatively framed items, the score is supposed to be subtracted by 5. Finally, scores are summed and multiplied by 2.5; the total usability score ranges from 0 to 100. A score of <50 was considered unacceptable, 50-70 was considered marginal, and >70 was considered acceptable (>85=excellent) [26].

#### Qualitative Interview

The interview questions were defined based on the research question and topics the study coordinators wanted to address, and their formulation followed basic guidelines for open-ended interview questions, such as omitting yes or no answers or leading questions [27]. Using a semistructured interview, the patient was interviewed for 15-30 minutes after the device had been used. Interviews were conducted in-person with audio recording, ensuring comprehensive capture of participant feedback. Audio-recorded interviews were transcribed verbatim by a professional transcription service using iflyrecClient.exe, with a 100% accuracy check performed by XY and PY to ensure fidelity to the original recordings. Transcripts were anonymized and stored securely in a password-protected database to protect participant confidentiality. The interview outline is detailed in Multimedia Appendix 4.

We used reflexive thematic analysis as described by Braun and Clarke [28], which is underpinned by a constructionist epistemology that recognizes themes as researcher-generated rather than emerging from data. Our analysis focused on shared meaning themes rather than topic summaries, seeking to understand the deeper experiences and perceptions of patients using the VR medical system. The analysis aimed to capture both semantic (explicit) and latent (implicit, interpretive) meanings within the data.

#### Adverse Events

Adverse reactions, characterization, severity, duration, and resolution will be recorded in detail for each use.

### Statistical Analyses

#### Quantitative Analysis

The total usability score was calculated using the original equation by Brooke [25]:



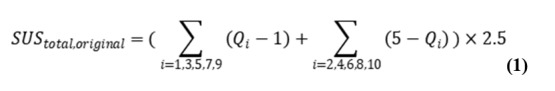



which results in a score between 0 (min) and 100 (max). For easier visual comparability between the individual SUS item ratings (Qi), we inverted the statements of negatively phrased items (Q2, Q4, Q6, Q8, and Q10) to make all statements positive and adapted the scoring accordingly [29]. The mean and SD of the question and the total usability score were calculated by SPSS Statistics (version 29.0; IBM Corp).

#### Qualitative Interview

Thematic analysis was conducted following Braun and Clarke’s [30] six-phase framework: (1) data familiarization, (2) initial code generation, (3) theme searching, (4) theme review, (5) theme definition and naming, and (6) report production [30]. Two researchers independently coded the transcripts using NVivo (version 15.0; QSR International), with regular meetings to discuss coding decisions and resolve discrepancies through consensus [29]. When no new themes emerged, the sample size was considered sufficient, and data saturation was achieved. Following Hennink and Kaiser’s [31] guidance, we used a code frequency counts strategy to assess data saturation. After each interview, transcripts were analyzed to identify new codes and themes. Data saturation was systematically assessed by tracking the emergence of new themes across sequential interviews.

To ensure the rigor of the qualitative findings, we followed Lincoln and Guba’s [32] criteria for trustworthiness: (1) credibility was enhanced through member checking, whereby key themes were reviewed by five participants for validation; (2) dependability was addressed by maintaining an audit trail documenting coding decisions and analytical processes; (3) confirmability was ensured through regular peer debriefings among the research team to minimize individual biases; (4) transferability was supported by providing rich, thick descriptions of participants and contexts; (5) reflexivity was maintained throughout, with the researchers engaging in ongoing self-reflection to consider how their backgrounds and preconceptions might influence the interpretation of data.

#### Mixed Methods Integration and Analysis

The steps for data integration were as follows: quantitative analysis, qualitative analysis, identification of similar and divergent findings, and confirmation, extension, or identification of discordant results [33]. Confirmation occurs when findings from both types of data reinforce each other. Extension is observed when divergent findings between the two datasets broaden insights into usability by addressing distinct or complementary aspects of the user experience. Discrepancy arises when survey and interview results are inconsistent, contradictory, or in disagreement with each other [23,34].

### Ethical Considerations

This study was classified as human participants research and was reviewed and approved by the Ethics Committee on Biomedical Research, West China Hospital of Sichuan University (1087). It was also registered as a clinical trial with the Chinese Clinical Trial Registry (ChiCTR2100051419). Written informed consent was obtained from all 37 participants prior to their enrollment, with consent forms explaining the study’s purpose, procedures, potential risks (eg, mild dizziness), and benefits. Participants were informed of their right to withdraw at any time without consequences. To protect privacy and confidentiality, each participant was assigned a unique numerical code, and all data (eg, SUS scores and interview transcripts) were deidentified and stored securely on password-protected files accessible only to the research team. Compensation was provided in the form of exemption from postoperative outpatient visit fees and 3-5 free postoperative nursing consultation services to support participants’ recovery. No images in the manuscript or supplementary materials (eg, Figure 1E) allow identification of individual participants, as facial features are obscured or not visible, ensuring compliance with privacy standards.

## Results

### Characteristics of the Participants

**Figure 2:**
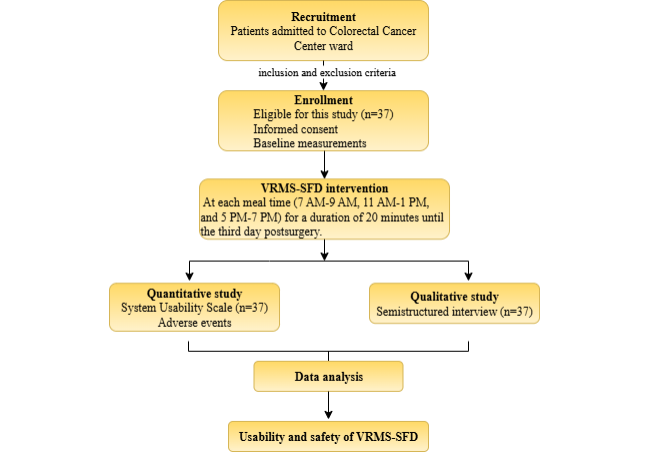
Flow diagram of a mixed methods study evaluating VRMS-SFD for patients undergoing colorectal cancer surgery at West China Hospital, Sichuan University, Chengdu, China, from 2022 to 2023, involving 37 postoperative patients with colorectal cancer, to assess the usability of a VRMS-SFD during perioperative fasting, including participant recruitment, intervention, and data collection processes. VRMS-SFD: virtual reality multisensory sham-feeding device.

A total of 37 participants were recruited for this study, and all completed the use of the VRMS-SFD. The flowchart for the participant inclusion process is shown in Figure 2. In the data collection stage, after 3 days of use, 37 questionnaires were collected, for a recovery rate of 100%. The mean age of the participants was 56.57 (SD 8.05) years; most of the participants were male (21/37, 57%), and all of them were married. The detailed characteristics of the participants are shown in Table 1 and can also be found in Multimedia Appendix 5.

**Table 1:** Characteristics of participants evaluating a VRMS-SFD^a^ for patients undergoing colorectal cancer surgery^b^.

Sample characteristics			Values (n=37)
**Sex, n (%)**			
	Male	21 (57)	
	Female	16 (43)	
Age (years), mean (SD)			56.57 (8.05)
Height (cm), mean (SD)			162.62 (9.34)
Weight (kg), mean (SD)			60.44 (10.46)
BMI (kg/m^2^), mean (SD)			22.82 (2.96)
**Nutritional status** ^c^ **, n (%)**			
	1	15 (41)	
	2	8 (22)	
	3	8 (22)	
	4	4 (11)	
	5	1 (3)	
	6	1 (3)	
**Education level, n (%)**			
	Primary school or under	9 (24)	
	Middle school	12 (32)	
	High school	6 (16)	
	University or above	10 (27)	
**Marital status, n (%)**			
	Married	37 (100)	
	Single	0 (0)	
	Divorced or widowed or else	0 (0)	
**Place of residence, n (%)**			
	City	19 (51)	
	Suburb	18 (49)	
**Medical insurance**			
	Yes	37 (100)	
	No	0 (0)	
**Have used VR** ^d^ **before**			
	Yes	0 (0)	
	No	37 (100)	

^a^VRMS-SFD: virtual reality multisensory sham-feeding device.

^b^None of the recruited patients had any previous experience with VR.

^c^The NRS2002 (Nutritional Risk Screening) scale was used to assess the nutritional status of participants, where lower scores signified lower nutritional risk and higher scores indicated higher nutritional risk.

^d^VR: virtual reality.

### Quantitative Results

**Figure 3:**
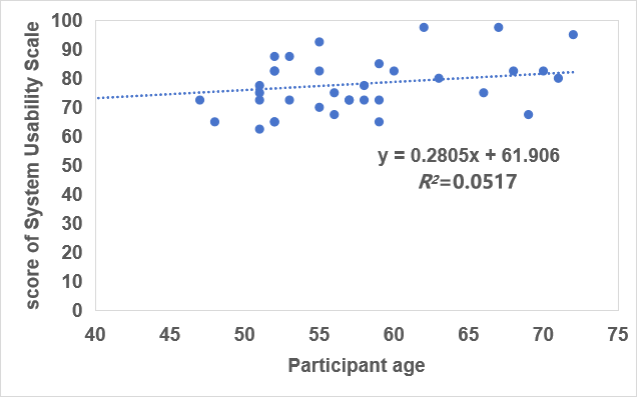
Correlation between age and System Usability Scale scores evaluating a virtual reality multisensory sham-feeding device for patients undergoing colorectal cancer surgery (n=37).

The usability of the VR feeding simulation device was assessed using the SUS, a 10-item questionnaire with scores ranging from 0 to 100. Across the 37 participants, the device achieved a mean SUS score of 77.78 (SD 7.90), with individual scores ranging from 62.5 to 97.5, indicating that the usability evaluation of this device is good, but there is still a need to improve it. The detailed scores for each item of the SUS are shown in Table 2 and can also be found in Multimedia Appendix 6. There was no significant correlation between participant age and usability score (Figure 3; *r*=0.05). Participants also rated their overall satisfaction with new technology using our device with a mean of 8.38 (SD 1.93) out of 10, indicating relative comfort navigating digital tools independently.

**Table 2:** System Usability Scale scores for a virtual reality multisensory sham-feeding device for patients undergoing colorectal cancer surgery.

Item No	Item	Score, mean (SD)^a^
Q1	I think that l would like to use this system frequently	4.11 (0.77)
Q2	I found the system unnecessarily complex	2.08 (0.86)
Q3	I thought the system was easy to use	4.46 (0.56)
Q4	I think that l would need the support of a technical person to be able to use this system	2.32 (0.78)
Q5	I found the various functions in this system were well integrated	4.05 (0.85)
Q6	I thought there was too much inconsistency in this system	1.81 (0.66)
Q7	I would imagine that most people would learn to use this system very quickly	4.27 (0.69)
Q8	I found the system very cumbersome to use	1.89 (0.77)
Q9	I felt very confident using the system	4.27 (0.61)
Q10	I needed to learn a lot of things before I could get going with this system	1.95 (0.70)

^a^Total score: mean 77.78 (SD 7.90).

### Qualitative Results

Qualitative data were collected through 15- to 30-minute semistructured interviews with 37 postoperative patients with colorectal cancer, to assess the usability and user experience of VRMS-SFD. Interviews were audio-recorded, transcribed verbatim in Mandarin. Two researchers independently coded the transcripts, achieving an intercoder agreement of 92%, and discrepancies were resolved through discussion with a third researcher. Data saturation was reached after 32 interviews, as no new themes emerged, but all 37 interviews were included to ensure comprehensive representation. Reflexivity was maintained by the research team documenting personal biases in reflective journals to minimize influence on analysis. Confirmability was ensured by maintaining an audit trail of coding decisions and raw data excerpts, linked to the identified themes (eg, immersive experience and reduced discomfort). Dependability was supported by providing detailed methodological descriptions. Credibility was enhanced through member checking, where five participants reviewed preliminary themes to verify accuracy, with minor clarifications incorporated. Transferability was addressed by providing thick descriptions of the study context, participant demographics, and clinical setting, enabling applicability to similar perioperative fasting contexts.

Thematic analysis yielded 4 major themes (overall perception of the device, improvement of perioperative fasting adverse effects, impact on appetite, and suggestions for follow-up improvement of VR equipment), 14 subthemes, and 203 nodes. Multimedia Appendix 7 presents the number of participants whose interviews included each subtheme. For instance, “Stimulates Appetite” was mentioned by 30 of 37 (81%) participants, while “Immersive Experience” was described by 13 of 37 (35%). Themes such as “Relieves Discomfort from Fasting” (20/37) and “Improves Mood” (9/37) showed considerable relevance to perceived benefit. Subthemes with low mention counts (eg, “Scent Too Strong” or “Add More Scenes”) reflect individualized user feedback and inform design iteration. Multimedia Appendix 7 provides the complete qualitative analysis, including themes, subthemes, and supporting participant quotes.

### Adverse Events

One of the 37 participants reported mild dizziness. This participant was a first-time user of VR equipment and experienced no associated symptoms such as nausea or vomiting. Upon the onset of dizziness, the VR device was promptly removed by the researchers, and the dizziness resolved within 5 minutes after device removal. Follow-up evaluations were conducted at 30 minutes, 24 hours, and 3 days postincident, with the participant reporting no further discomfort.

### Mixed Methods Results

The themes that emerged from the qualitative results were compared and merged with the quantitative results in terms of the overall perception of the device, the improvement of perioperative fasting adverse effects, the impact on appetite, and suggestions for the follow-up improvement of our device. Quantitative data showed that the overall usability of our device was good, especially scores on item 3 (I think the system was easy to use), item 6 (I thought there was too much inconsistency in this system), but some aspects still need further improvement. Specific usability issues (overall experience, its effectiveness in reducing discomfort during preoperative fasting, its impact on appetite, and suggestions) of the VRMS-SFD were deeply explored through qualitative interviews. The overall experience of the device was excellent, immersive, and enjoyable. As for discomfort during perioperative fasting, our device relieved the discomfort of being unable to eat, improved perioperative mood, and promoted intestinal peristalsis. It also stimulated appetite. The optimization suggestions included calibration of scents, simplifying operations, and improving background music. Quantitative results and qualitative results were generally consistent, and no discordant results were observed. The comparison and merging of the 2 datasets resulted in confirmed and expanded findings.

## Discussion

### Principal Findings

This mixed methods study evaluated the usability, acceptability, and safety of the VRMS-SFD in 37 postoperative patients with colorectal cancer during perioperative fasting. The mean usability score of the device was 77.78 (SD 7.90), above the suggested cut-off value of 70, indicating considered acceptable usability and was graded as B+ [25,26]. According to our usability assessment, item 3 (mean 4.46, SD 0.56) and item 10 (mean 1.95, SD 0.70) indicated easy learnability of our equipment, whereas item 9 (mean 4.21, SD 0.67), item 1 (mean 4.11, SD 0.67), and item 5 (mean 4.05, SD 0.85) demonstrated confidence and general usability among participants [35,36]. This is also supported by comments in the qualitative interviews, where most feedback was positive.

Qualitative analysis produced four principal themes: overall perception of the device, improvement of perioperative fasting adverse effects, impact on appetite, and suggestions for follow-up improvement of VR equipment. Thematic coding achieved an intercoder agreement of 92%; data saturation was reached after 32 interviews, and all 37 interviews were analyzed to ensure comprehensive representation. Participants commonly described the device as “novel” and “enjoyable,” reporting perceived benefits such as improved mood, reduced bloating or discomfort, reports of increased peristalsis or gas passage, and enhanced appetite. Participants also provided actionable feedback, including improving scent authenticity, simplifying controls, and expanding food selections.

Safety findings were favorable: one participant experienced transient dizziness that resolved within 5 minutes after device removal and postural adjustment; no other adverse events were observed. Overall, the quantitative and qualitative results converged to indicate the feasibility, acceptability, and preliminary patient-perceived benefits of the VRMS-SFD in the studied perioperative context.

### Comparison to Prior Work

The majority of participants felt that the device offered an immersive experience, describing it as both “novel” and “enjoyable.” This could be attributed to the difference in both form and content between the VRMS-SFD and conventional interventions (eg, medication and injections). By integrating a series of technologies, VR technology provides a multisensory and 3D environments that enable users to become fully immersed in a simulated world, and its immersive property makes the patients actively interact with the vivid VR environment, which demands more attention [37]. Similar to this study, the VR app developed by Langlet et al [38] for treating eating behavior in patients with eating disorders (ED) received an average score of 73.4 (SD 9.2; range 55-90) and was rated as “good” overall. However, due to being in the early stage of development, the app’s usability was evaluated by clinicians, physicians (who have daily interactions with patients with ED), and IT personnel, rather than by patients with ED themselves.

In addition, the usability of our device does not appear to be affected by age. Previous studies have shown that younger users are more receptive to VR environments [39,40]; however, in this study, no significant correlation between participant age and usability score (*r*=0.05) was observed. The consistent ease of use of our device across different age groups indicated good universality.

With regard to its clinical effect, some participants indicated that the VRMS-SFD can ease discomfort during perioperative fasting. On the one hand, some participants reported that our device helped improve their mood. As a distraction tool, VR has proven effective in clinical settings, with multiple studies demonstrating its ability to significantly alleviate pain and reduce anxiety in perioperative medical contexts [41,42]. A number of participants highlighted that our device could free them from the frustration of being unable to eat. A likely explanation could be that the multiple stimuli during device use activated the orbitofrontal cortex, which led to an enhanced hedonic response and increased pleasantness [43]. On the other hand, patients also perceived it to have a positive effect in promoting the recovery of gastrointestinal function, such as relieving bloating, promoting intestinal peristalsis, and facilitating gas expulsion. This could be explained from the perspective of neurogastroenterology. The gut-brain axis is the bidirectional communication system between the gastrointestinal tract and the central nervous system [44]. Gastrointestinal motility is regulated by the enteric nervous system and extrinsic factors, including the brain, the autonomic nervous system, the gut-associated immune system, and the gut microbiome [45]. These factors work together to modulate gastrointestinal function and maintain homeostasis. Modulation of intrinsic enteric neuron activity via top-down signals or microbe-associated molecules ultimately influences gastrointestinal physiology [46]. In response to stimuli like the smell and sight of food, hormones that prepare for food consumption—including insulin, ghrelin, gastrin, glucagon-like peptide-1, and cholecystokinin—are released [47]. This is the so-called cephalic response, which is one of the most common forms of conditioning. Some of these pancreatic and gastric secretions exert a stimulatory effect on the gastrointestinal motility [48]. As a result, many participants shared that the device boosted their appetite to some extent. Appetite loss is a pervasive and quality-of-life–altering problem after major abdominal surgery [49]; 55% patients reported decreased appetite after colorectal surgery [50]. Postoperative loss of appetite may lead to weight loss and impaired intestinal absorption, which can subsequently result in malnutrition, adversely affect patients’ survival, quality of life, and increase the risk of complications [51,52]. The abdominal discomfort of patients was relieved to some degree. This could be the primary factor contributing to the recognition of the VRMS-SFD among participants.

Several studies have shown that VR-enhanced cognitive behavioral therapy can regulate the appetite and eating frequency of patients with ED [53], highlighting the potential of VR in addressing such problems. Liem et al [54] proposed that food choices were influenced by the sensory input from the environment. Ferrer-Garcia et al [55] conducted VR-based cue exposure therapy that included a library of 30 most craved VR foods among participants and 4 real-life VR environments, aiming to diminish food-related anxiety and cravings, thereby reducing binge eating. Ludovica exposed patients with anorexia nervosa to a VR kitchen environment to reduce anxiety around eating and food avoidance. Previous studies also suggested that the involvement of multiple senses can enhance the feeling of presence and associated food desire [54,56]. However, studies similar to the aforementioned ones have typically been limited to visual cues, with only occasional use of sound or music in the VR environment. In contrast, this study used synchronized visual, auditory, and olfactory modalities associated with food, creating an immersive VR dining experience for participants that effectively aroused their appetite.

Methodologically, our mixed methods design is consistent with contemporary digital-health usability research. Lo et al [57] suggested that the use of a mixed method design could contribute to a better understanding of the intervention. The National Institutes of Health Office of Behavioral and Social Science Research also recommends the application of mixed methods in the evaluation of new instruments or methods, since it offers a comprehensive understanding by combining qualitative and quantitative data. In this way, it fosters innovation, supports the development and refinement of tools, and enhances the scientific rigor and practical applicability of findings [58]. This mixed methods usability study provided evidence on the application of the VRMS-SFD among patients with colorectal cancer who are undergoing surgery. The integration of quantitative metrics and qualitative feedback offers an understanding of both the device’s technical performance and its acceptance within the clinical context. These findings reveal several key aspects regarding the feasibility and potential therapeutic value of VR–based feeding intervention in postoperative care.

### Limitations

First, this study evaluated short-term usability as a single phase within an iterative design process. While this allowed for focused feedback on initial device performance, it also limits conclusions regarding long-term clinical outcomes. We attempted to mitigate this by integrating both quantitative usability scores and qualitative feedback, ensuring a comprehensive assessment of early feasibility. Nevertheless, longitudinal studies are needed to confirm the durability of effects and assess whether identified usability issues can be resolved through subsequent design iterations.

Second, the study sample was relatively small and drawn exclusively from a single medical institution, which may introduce selection bias and limit generalizability. To reduce this risk, we recruited consecutive eligible patients within the defined study period and ensured demographic diversity within the cohort. However, expanding future trials to include larger and multicenter populations will be essential to enhance the external validity and representativeness of findings.

Third, this study relied heavily on self-reported data, which are susceptible to subjective bias. While triangulation through mixed methods (quantitative SUS scores and qualitative thematic analysis) improved credibility, the absence of objective physiological markers restricts the interpretation of the biological impact of the intervention. Future studies should incorporate measures such as gastrointestinal motility monitoring, abdominal circumference, and serum biomarkers of the gut-brain axis (eg, ghrelin, leptin, and cholecystokinin), which would provide more robust evidence and strengthen the confirmability and dependability of results.

Fourth, one participant reported experiencing dizziness during the VR session. Since individuals with prior symptoms of dizziness, vestibular disease, or cybersickness were excluded beforehand, the occurrence of dizziness in this case could be attributed to factors such as residual effect of anesthesia, metabolic disorder due to perioperative fasting, adverse effects of postoperative analgesics, or reduced cerebral blood flow caused by abrupt position changes after prolonged immobility postsurgery [59-61]. Besides, mild anxiety might also have played a contributing role [62]. To mitigate such adverse events in the future, sufficient recovery from anesthesia, gradual postural adjustment, and careful monitoring should be prioritized.

### Future Directions

Participants in this study actively engaged in suggesting ways to improve the VRMS-SFD, highlighting both technical and experiential aspects. They identified issues such as the limited realism of certain food models and proposed practical enhancements, including more precise calibration of olfactory stimuli and adjustments to the background music to create a more immersive and enjoyable environment. These insights provide a valuable foundation for iterative design and patient-centered refinement.

Looking ahead, future development should prioritize incorporating patient feedback into systematic device upgrades, expanding the diversity of VR dining scenarios, and integrating adaptive features that tailor sensory input to individual preferences or clinical conditions. Beyond technical improvements, advancing this work will require sustained multidisciplinary collaboration among clinicians, engineers, psychologists, and designers. Such cooperation will be essential to optimize usability, enhance ecological validity, and ultimately develop the next generation of multisensory VR sham-feeding interventions with broader clinical applicability.

### Conclusions

This mixed methods study evaluated the usability, acceptability, and safety of the VRMS-SFD among patients with colorectal cancer during perioperative fasting. Quantitative findings demonstrated a mean SUS score of 77.78 (SD 7.90), exceeding the threshold for acceptable usability, with consistently high ratings across age groups. Qualitative interviews confirmed these results, revealing that patients perceived the device as novel, engaging, and effective in alleviating fasting-related discomfort and stimulating appetite. Safety outcomes were favorable, with only one transient and self-limited adverse event reported. Taken together, these findings indicate that the VRMS-SFD may be a feasible and well-accepted tool for supporting patient comfort and appetite regulation during perioperative fasting, thereby addressing a critical unmet need in colorectal cancer care.

## Appendix

Multimedia Appendix 1 MMR-RHS-checklist.

Multimedia Appendix 2 Intervention process.

Multimedia Appendix 3 VRMS-SFD patient demonstration video.

Multimedia Appendix 4 Patient questionnaires.

Multimedia Appendix 5 Characteristics of participants evaluating a virtual reality multisensory sham-feeding device for colorectal cancer surgery patients (n=37). None of the recruited patients had any previous experience with virtual reality.

Multimedia Appendix 6 System Usability Scale (SUS) scores for a virtual reality multisensory sham-feeding device of colorectal cancer surgery patients.

Multimedia Appendix 7 Results of the interview content analysis.

## Abbreviations

ED: eating disorder
IDEAS: Integrate, Design, Assess, and Share
MMR-RHS: Mixed Methods Reporting in Rehabilitation and Health Sciences
SUS: System Usability Scale
VR: virtual reality
VRMS-SFD: virtual reality multisensory sham-feeding device

## Data Availability

The datasets generated or analyzed during this study are not publicly available due to privacy restrictions and institutional policies on patient data sharing, but are available from the corresponding author on reasonable request.

## References

[1] Bray F, Laversanne M, Sung H, Ferlay J, Siegel RL, Soerjomataram I, Jemal A (2024). Global cancer statistics 2022: GLOBOCAN estimates of incidence and mortality worldwide for 36 cancers in 185 countries. CA Cancer J Clin.

[2] Vogel JD, Felder SI, Bhama AR, Hawkins AT, Langenfeld SJ, Shaffer VO, Thorsen AJ, Weiser MR, Chang GJ, Lightner AL, Feingold DL, Paquette IM (2022). The American Society of Colon and Rectal Surgeons clinical practice guidelines for the management of colon cancer. Dis Colon Rectum.

[3] Joshi GP, Abdelmalak BB, Weigel WA, Harbell MW, Kuo CI, Soriano SG, Stricker PA, Tipton T, Grant MD, Marbella AM, Agarkar M, Blanck JF, Domino KB (2023). 2023 American Society of anesthesiologists practice guidelines for preoperative fasting: carbohydrate-containing clear liquids with or without protein, chewing gum, and pediatric fasting duration-a modular update of the 2017 American society of anesthesiologists practice guidelines for preoperative fasting. Anesthesiology.

[4] Hirota K (2021). Hypoxia-dependent signaling in perioperative and critical care medicine. J Anesth.

[5] Nagai M, Noguchi R, Takahashi D, Morikawa T, Koshida K, Komiyama S, Ishihara N, Yamada T, Kawamura YI, Muroi K, Hattori K, Kobayashi N, Fujimura Y, Hirota M, Matsumoto R, Aoki R, Tamura-Nakano M, Sugiyama M, Katakai T, Sato S, Takubo K, Dohi T, Hase K (2019). Fasting-refeeding impacts immune cell dynamics and mucosal immune responses. Cell.

[6] Sommer NP, Schneider R, Wehner S, Kalff JC, Vilz TO (2021). State-of-the-art colorectal disease: postoperative ileus. Int J Colorectal Dis.

[7] Bragg D, El-Sharkawy AM, Psaltis E, Maxwell-Armstrong CA, Lobo DN (2015). Postoperative ileus: recent developments in pathophysiology and management. Clin Nutr.

[8] Hill A, Heyland DK, Reyes LAO, Laaf E, Wendt S, Elke G, Stoppe C (2022). Combination of enteral and parenteral nutrition in the acute phase of critical illness: an updated systematic review and meta-analysis. JPEN J Parenter Enteral Nutr.

[9] Willner A, Teske C, Hackert T, Welsch T (2023). Effects of early postoperative mobilization following gastrointestinal surgery: systematic review and meta-analysis. BJS Open.

[10] Tang G, Huang W, Tao J, Wei Z (2022). Prophylactic effects of probiotics or synbiotics on postoperative ileus after gastrointestinal cancer surgery: a meta-analysis of randomized controlled trials. PLoS One.

[11] Feldman M, Richardson CT (1986). Role of thought, sight, smell, and taste of food in the cephalic phase of gastric acid secretion in humans. Gastroenterology.

[12] Li K, Yuan X, Hu Y, Zhang W, Chen Y, Hong R, Yang J (2023). [An integrated audio-visual-olfactory virtual reality false feeding device: research, development, and design]. Sichuan Da Xue Xue Bao Yi Xue Ban.

[13] Sammut R, Trapani J, Deguara J, Ravasi V (2021). The effect of gum chewing on postoperative ileus in open colorectal surgery patients: a review. J Perioper Pract.

[14] Liu Q, Jiang H, Xu D, Jin J (2017). Effect of gum chewing on ameliorating ileus following colorectal surgery: a meta-analysis of 18 randomized controlled trials. Int J Surg.

[15] Jallad ST, Işık B (2022). The effectiveness of virtual reality simulation as learning strategy in the acquisition of medical skills in nursing education: a systematic review. Ir J Med Sci.

[16] Zhong D, Chen L, Feng Y, Song R, Huang L, Liu J, Zhang L (2021). Effects of virtual reality cognitive training in individuals with mild cognitive impairment: a systematic review and meta-analysis. Int J Geriatr Psychiatry.

[17] Turgut A, İlçe AO, Öztürk H (2024). The effect of immersive virtual reality application on anxiety, pain, and parental satisfaction in the perioperative process of children: a randomized controlled trial. Pain Manag Nurs.

[18] Kiper P, Szczudlik A, Agostini M, Opara J, Nowobilski R, Ventura L, Tonin P, Turolla A (2018). Virtual reality for upper limb rehabilitation in subacute and chronic stroke: a randomized controlled trial. Arch Phys Med Rehabil.

[19] Huang L-C, Chien C-F, Yang Y-H (2025). Exploring the immediate and long-term effects of immersive virtual reality on behavioral and psychological symptoms of dementia and caregiver burden: longitudinal observational study. JMIR Serious Games.

[20] Sauchelli S, Brunstrom JM (2022). Virtual reality exergaming improves affect during physical activity and reduces subsequent food consumption in inactive adults. Appetite.

[21] Xia W, Ding J, Yan Y, Chen F, Yan M, Xu X (2024). Effectiveness of virtual reality technology in symptom management of patients at the end of life: a systematic review and meta-analysis. J Am Med Dir Assoc.

[22] Mummah SA, Robinson TN, King AC, Gardner CD, Sutton S (2016). IDEAS (Integrate, Design, Assess, and Share): a framework and toolkit of strategies for the development of more effective digital interventions to change health behavior. J Med Internet Res.

[23] Leng M, Sun Y, Li C, Han S, Wang Z (2023). Usability evaluation of a knowledge graph-based dementia care intelligent recommender system: mixed methods study. J Med Internet Res.

[24] Tovin MM, Wormley ME (2023). Systematic development of standards for mixed methods reporting in rehabilitation health sciences research. Phys Ther.

[25] Brooke J, Jordan PW, Thomas B, Weerdmeester BA, McClelland IL (1996). SUS—a quick and dirty usability scale. Usability Evaluation in Industry.

[26] Bangor A, Kortum P, Miller J (2009). Determining what individual SUS scores mean: adding an adjective rating scale. J Usability Stud.

[27] Wilson C (2013). Interview Techniques for UX Practitioners: A User-Centered Design Method.

[28] Braun V, Clarke V (2023). Is thematic analysis used well in health psychology? A critical review of published research, with recommendations for quality practice and reporting. Health Psychol Rev.

[29] Dittli J, Meyer JT, Gantenbein J, Bützer T, Ranzani R, Linke A, Curt A, Gassert R, Lambercy O (2023). Mixed methods usability evaluation of an assistive wearable robotic hand orthosis for people with spinal cord injury. J Neuroeng Rehabil.

[30] Braun V, Clarke V (2008). Using thematic analysis in psychology. Qual Res Psychol.

[31] Hennink M, Kaiser BN (2022). Sample sizes for saturation in qualitative research: a systematic review of empirical tests. Soc Sci Med.

[32] Lincoln YS, Guba EG (1986). But is it rigorous? Trustworthiness and authenticity in naturalistic evaluation. New Dir Program Eval.

[33] Creswell JW, Clark VLP (2017). Designing and Conducting Mixed Methods Research.

[34] Fetters MD, Curry LA, Creswell JW (2013). Achieving integration in mixed methods designs-principles and practices. Health Serv Res.

[35] Borsci S, Federici S, Lauriola M (2009). On the dimensionality of the System Usability Scale: a test of alternative measurement models. Cogn Process.

[36] Lewis JR, Sauro JF (2009). The factor structure of the System Usability Scale.

[37] Li L, Yu F, Shi D, Shi J, Tian Z, Yang J, Wang X, Jiang Q (2017). Application of virtual reality technology in clinical medicine. Am J Transl Res.

[38] Langlet BS, Odegi D, Zandian M, Nolstam J, Södersten Per, Bergh C (2021). Virtual reality app for treating eating behavior in eating disorders: development and usability study. JMIR Serious Games.

[39] Lorenz M, Brade J, Klimant P, Heyde C, Hammer N (2023). Age and gender effects on presence, user experience and usability in virtual environments-first insights. PLoS One.

[40] Coxon M, Kelly N, Page S (2016). Individual differences in virtual reality: are spatial presence and spatial ability linked?. Virtual Reality.

[41] Chiu PL, Li H, Yap KY, Lam KC, Yip PR, Wong CL (2023). Virtual reality-based intervention to reduce preoperative anxiety in adults undergoing elective surgery: a randomized clinical trial. JAMA Netw Open.

[42] El Mathari S, Shehadeh S, Zwaan WP, Boulidam N, Kuitert L, Twisk JWR, Klautz RJM, de Lind van Wijngaarden R, Veen K, Kluin J (2024). The effect of virtual reality on postoperative anxiety and pain in patients following cardiac surgery: a randomized controlled trial. Eur J Cardiothorac Surg.

[43] Spence C (2015). Multisensory flavor perception. Cell.

[44] Mayer EA, Knight R, Mazmanian SK, Cryan JF, Tillisch K (2014). Gut microbes and the brain: paradigm shift in neuroscience. J Neurosci.

[45] Margolis KG, Cryan JF, Mayer EA (2021). The microbiota-gut-brain axis: from motility to mood. Gastroenterology.

[46] Agirman G, Hsiao EY (2021). SnapShot: the microbiota-gut-brain axis. Cell.

[47] Skvortsova A, Veldhuijzen DS, Kloosterman IE, Pacheco-López G, Evers AW (2021). Food anticipatory hormonal responses: a systematic review of animal and human studies. Neurosci Biobehav Rev.

[48] Mori H, Verbeure W, Schol J, Carbone F, Tack J (2022). Gastrointestinal hormones and regulation of gastric emptying. Curr Opin Endocrinol Diabetes Obes.

[49] Wagner M, Probst P, Haselbeck-Köbler M, Brandenburg JM, Kalkum E, Störzinger D, Kessler J, Simon JJ, Friederich H, Angelescu M, Billeter AT, Hackert T, Müller-Stich BP, Büchler MW (2022). The problem of appetite loss after major abdominal surgery: a systematic review. Ann Surg.

[50] Wennström B, Johansson A, Kalabic S, E-Son Loft A-L, Skullman S, Bergh I (2020). Patient experience of health and care when undergoing colorectal surgery within the ERAS program. Perioper Med (Lond).

[51] Nguyen TH, Ta NT, Dang AK, Nguyen TT, Dam VAT, Latkin CA, Ho CSH, Ho RCM (2023). A longitudinal assessment of appetite loss and nutritional care among postoperative patients in Vietnam. Front Nutr.

[52] Martin L, Lagergren P (2015). Risk factors for weight loss among patients surviving 5 years after esophageal cancer surgery. Ann Surg Oncol.

[53] Ferrer-García M, Gutiérrez-Maldonado J, Pla-Sanjuanelo J, Vilalta-Abella F, Riva G, Clerici M, Ribas-Sabaté J, Andreu-Gracia A, Fernandez-Aranda F, Forcano L, Riesco N, Sánchez I, Escandón-Nagel N, Gomez-Tricio O, Tena V, Dakanalis A (2017). A randomised controlled comparison of second-level treatment approaches for treatment-resistant adults with bulimia nervosa and binge eating disorder: assessing the benefits of virtual reality cue exposure therapy. Eur Eat Disord Rev.

[54] Liem DG, Mawas M, Keast RS (2023). Evoked sensory stimulation of the eating environment, impacts feeling of presence and food desires in an online environment. Food Res Int.

[55] Ferrer-Garcia M, Pla-Sanjuanelo J, Dakanalis A, Vilalta-Abella F, Riva G, Fernandez-Aranda F, Forcano L, Riesco N, Sánchez I, Clerici M, Ribas-Sabaté J, Andreu-Gracia A, Escandón-Nagel N, Gomez-Tricio O, Tena V, Gutiérrez-Maldonado J (2019). A randomized trial of virtual reality-based cue exposure second-level therapy and cognitive behavior second-level therapy for bulimia nervosa and binge-eating disorder: outcome at six-month followup. Cyberpsychol Behav Soc Netw.

[56] Marucci M, Di Flumeri G, Borghini G, Sciaraffa N, Scandola M, Pavone EF, Babiloni F, Betti V, Aricò P (2021). The impact of multisensory integration and perceptual load in virtual reality settings on performance, workload and presence. Sci Rep.

[57] Lo HHM, Ng M, Fong PYH, Lai HHK, Wang B, Wong SY, Sit RWS (2024). Examining the feasibility, acceptability, and preliminary efficacy of an immersive virtual reality-assisted lower limb strength training for knee osteoarthritis: mixed methods pilot randomized controlled trial. JMIR Serious Games.

[58] Creswell JW, Klassen AC, Plano CVL, Smith KC (2013). Best Practices for Mixed Methods Research in the Health Sciences.

[59] Bennett J, McDonald T, Lieblich S, Piecuch J (1999). Perioperative rehydration in ambulatory anesthesia for dentoalveolar surgery. Oral Surg Oral Med Oral Pathol Oral Radiol Endod.

[60] Seok Y, Suh EE, Yu S, Park J, Park H, Lee E (2021). Effectiveness of integrated education to reduce postoperative nausea, vomiting, and dizziness after abdominal surgery under general anesthesia. Int J Environ Res Public Health.

[61] Li Y, Dou Z, Yang L, Wang Q, Ni J, Ma J (2021). Oxycodone versus other opioid analgesics after laparoscopic surgery: a meta-analysis. Eur J Med Res.

[62] Huang J, Lin J, Xiong Y, Wang Z, Zhu Y, Ye H, Guo W (2020). Risk factors associated with postoperative discomfort after ambulatory strabismus surgery under general anesthesia. J Pain Res.

