# Supplementary material for: Usability Evaluation of a Virtual Reality Multisensory Sham-Feeding Device for Patients Undergoing Fasting Periods for Colorectal Cancer Surgery: Mixed Methods Study
Source: JMIR Serious Games. 2025 Oct 8;13:e75641. doi: 10.2196/75641 (PMC12547343; doi:10.2196/75641)
Supplement: Multimedia Appendix 2 [file games_v13i1e75641_app2.docx]

**Intervention Process**

1. Evaluation and Preparation of Multi-Sensory Stimulation Sham Feeding VR Equipment:
2. Confirm the patient's bed number, name, and hospitalization number,
3. Assess the patient's vital signs, level of consciousness, and check for any bleeding from the surgical site to ensure safety and comfort for VR usage,
4. Evaluate the patient’s cognitive understanding and cultural background to determine suitability for VR equipment, Ensure the patient or guardian has signed the consent form for VR equipment usage,
5. Verify that the VR headset, aroma emitter, operating handle, and other hardware are fully charged, have a normal network connection, and are functioning properly.
6. Patient Engagement with VR
7. Assist patients in donning the VR headset and aroma emitter, guiding them in the correct use of the operating handle,
8. Navigate the patient through a virtual food street, allowing selection of a Chinese restaurant (a), fruit shop (b), or dessert shop (c),


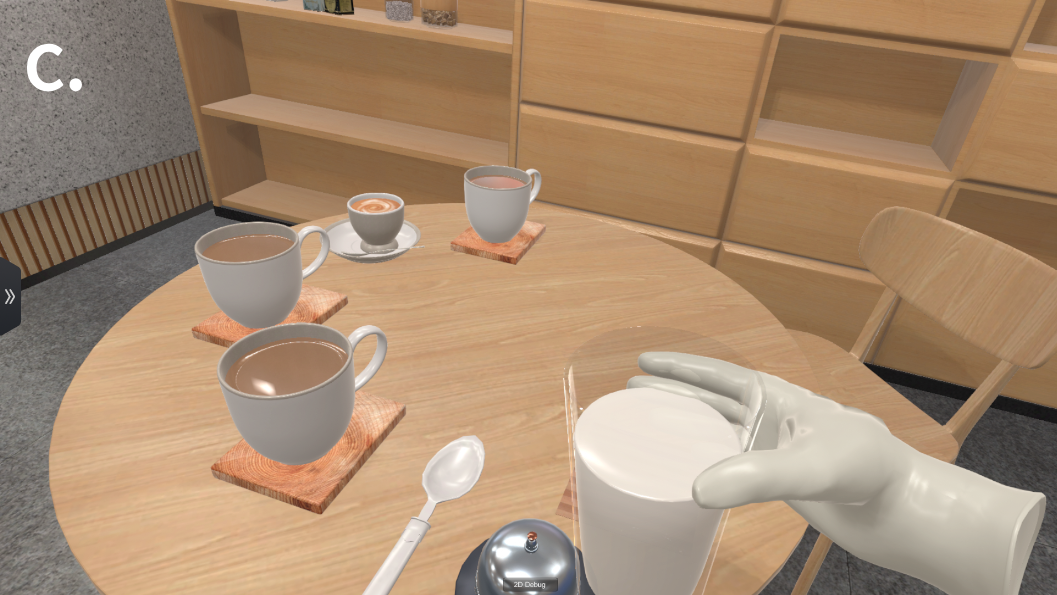

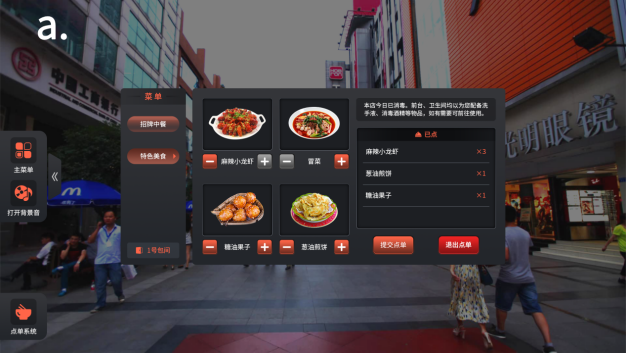

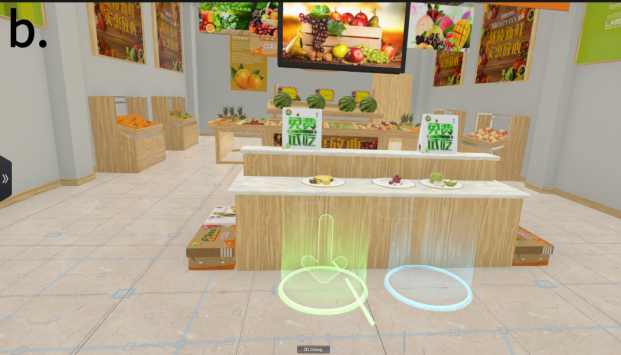


1. Enter the virtual restaurant setting and assist in seating the patient through the handle,
2. Facilitate communication with the virtual waiter while enabling interaction through the head-mounted device; assist in selecting dishes from the menu by clicking with the handle,
3. Display the dish preparation and presentation process through video, animation, or three-dimensional action on the head-mounted device,
4. Enable the patient to initiate the dining experience by clicking on tableware and food, simulating the eating process through handle or gesture interaction, while the aroma emitter emits the corresponding food aromas,
5. Continue the experience until the meal is completed.
6. Equipment Maintenance:
7. Post-use, switch off the equipment, disinfect with alcohol wipes, and replace the consumed virtual aroma emitter.
