# Supplementary material for: Usability Evaluation of a Virtual Reality Multisensory Sham-Feeding Device for Patients Undergoing Fasting Periods for Colorectal Cancer Surgery: Mixed Methods Study
Source: JMIR Serious Games. 2025 Oct 8;13:e75641. doi: 10.2196/75641 (PMC12547343; doi:10.2196/75641)
Supplement: Multimedia Appendix 4 [file games_v13i1e75641_app4.docx]

Dear Participant,

Thank you for taking part in this study. This study aims to evaluate the usability and patient experience of a Virtual Reality Multi-Sensory Sham-Feeding Device in colorectal cancer patients during the perioperative period. Your responses will help us improve device design and enhance clinical care. Please answer the following questionnaire truthfully based on your experience. All information will be kept strictly confidential and used solely for research purposes. We sincerely appreciate your participation and support.

1. **Basic information**

Please complete the following basic information. This will help us understand how patient backgrounds influence user experience.

1. Gender: □male □female
2. Age:
3. Height : cm
4. Weight : kg
5. Education level: □ Primary school or under □ Middle school □ High school □ University or above
6. Marital status: □ Married □ Single □ Divorced/Widowed/Else
7. Place of residence: □ City □Suburb
8. Medical insurance: □ yes □no
9. Have you used VR before？ □ yes □no
10. **System Usability Scale (SUS)**

This section assesses the usability of the device. Based on your experience, please select the option that best reflects your opinion for each item (1 = Strongly disagree, 2 = Disagree, 3 = Neutral or unsure, 4 = Agree, 5 = Strongly agree.).

| **Item No** | **Item** | **Strongly**  **disagree** | **Disagree** | **Neutral** | **Agree** | **Strongly**  **agree** |
| --- | --- | --- | --- | --- | --- | --- |
| Q1 | I think that l would like to use this system frequently. | 1 | 2 | 3 | 4 | 5 |
| Q2 | I found the system unnecessarily complex. | 1 | 2 | 3 | 4 | 5 |
| Q3 | I thought the system was easy to use. | 1 | 2 | 3 | 4 | 5 |
| Q4 | I think that l would need the support of a technical person to be able to use this system. | 1 | 2 | 3 | 4 | 5 |
| Q5 | I found the various functions in this system were well integrated. | 1 | 2 | 3 | 4 | 5 |
| Q6 | I thought there was too much inconsistency in this system. | 1 | 2 | 3 | 4 | 5 |
| Q7 | I would imagine that most people would learn to use this system very quickly. | 1 | 2 | 3 | 4 | 5 |
| Q8 | I found the system very cumbersome to use. | 1 | 2 | 3 | 4 | 5 |
| Q9 | I felt very confident using the system. | 1 | 2 | 3 | 4 | 5 |
| Q10 | I needed to learn a lot of things before l could get going with this system. | 1 | 2 | 3 | 4 | 5 |

1. **interview guide:**

These are open-ended interview questions that will be discussed in person by the researcher to gain deeper insights into your experience and suggestions.

1、Your overall experience after using this VR device

2、How do you think the device affected your discomfort caused during perioperative fasting?

3、How do you think the device affects your appetite?

4、How would you recommend this device?

1. **Adverse events**

Please record any adverse reactions experienced during device use, including details of the event and resolution.

| Adverse reactions | Whether it happens | severity | When it happens | duration | resolution | treatment efficiency |
| --- | --- | --- | --- | --- | --- | --- |
|  |  |  |  |  |  |  |
|  |  |  |  |  |  |  |
