# Supplementary material for: Usability Evaluation of a Virtual Reality Multisensory Sham-Feeding Device for Patients Undergoing Fasting Periods for Colorectal Cancer Surgery: Mixed Methods Study
Source: JMIR Serious Games. 2025 Oct 8;13:e75641. doi: 10.2196/75641 (PMC12547343; doi:10.2196/75641)
Supplement: Multimedia Appendix 5 [file games_v13i1e75641_app5.docx]

| **Sample characteristics** | **Values** |
| --- | --- |
| **Sex, n (%)** |  |
| Male | 21 (56.76) |
| Female | 16 (43.24) |
| **Age (years), mean (SD)** | 56.57 (8.05) |
| **Height (cm), mean (SD)** | 162.62 (9.34) |
| **Weight (kg), mean (SD)** | 60.44 (10.46) |
| **BMI (kg/m^2^)** | 22.82 (2.96) |
| **Nutritional status*, n (%)** |  |
| 1 | 15 (40.54) |
| 2 | 8 (21.62) |
| 3 | 8 (21.62) |
| 4 | 4 (10.81) |
| 5 | 1 (2.70) |
| 6 | 1 (2.70) |
| **Education level, n (%)** |  |
| Primary school or under | 9 (24.32) |
| Middle school | 12 (32.43) |
| High school | 6 (16.22) |
| University or above | 10 (27.03) |
| **Marital status, n (%)** |  |
| Married | 37 (100) |
| Single | 0 (0) |
| Divorced/Widowed/Else | 0 (0) |
| **Place of residence, n (%)** |  |
| City | 19 (51.35) |
| Suburb | 18 (48.65) |
| **Medical insurance, n (%)** |  |
| Yes | 37 (100) |
| No | 0 (0) |
| **Have used VR before, n (%)** |  |
| Yes | 0 (0) |
| No | 37 (100) |

*The NRS2002 (Nutritional Risk Screening) scale was employed to assess the nutritional status of participants, where lower scores signified lower nutritional risk and higher scores indicated higher nutritional risk.
