# Supplementary material for: Usability Evaluation of a Virtual Reality Multisensory Sham-Feeding Device for Patients Undergoing Fasting Periods for Colorectal Cancer Surgery: Mixed Methods Study
Source: JMIR Serious Games. 2025 Oct 8;13:e75641. doi: 10.2196/75641 (PMC12547343; doi:10.2196/75641)
Supplement: Multimedia Appendix 6 [file games_v13i1e75641_app6.docx]

| **Item No** | **Item** | **Score, mean (SD)** |
| --- | --- | --- |
| Q1 | I think that l would like to use this system frequently. | 4.11 (0.77) |
| Q2 | I found the system unnecessarily complex. | 2.08 (0.86) |
| Q3 | I thought the system was easy to use. | 4.46 (0.56) |
| Q4 | I think that l would need the support of a technical person to be able to use this system. | 2.32 (0.78) |
| Q5 | I found the various functions in this system were well integrated. | 4.05 (0.85) |
| Q6 | I thought there was too much inconsistency in this system. | 1.81 (0.66) |
| Q7 | I would imagine that most people would learn to use this system very quickly. | 4.27 (0.69) |
| Q8 | I found the system very cumbersome to use. | 1.89 (0.77) |
| Q9 | I felt very confident using the system. | 4.27 (0.61) |
| Q10 | I needed to learn a lot of things before l could get going with this system. | 1.95 (0.70) |
|  | Total system usability scale score | 77.78 (7.90) |
