# Supplementary material for: Usability Evaluation of a Virtual Reality Multisensory Sham-Feeding Device for Patients Undergoing Fasting Periods for Colorectal Cancer Surgery: Mixed Methods Study
Source: JMIR Serious Games. 2025 Oct 8;13:e75641. doi: 10.2196/75641 (PMC12547343; doi:10.2196/75641)
Supplement: Multimedia Appendix 7 [file games_v13i1e75641_app7.docx]

| **Theme** | **Subtheme** | **Number of nodes** | **Authors’ summary** | **Participants’ Quotes** |
| --- | --- | --- | --- | --- |
| Overall Experience | Excellent | 31 | Based on patient feedback, overall, they expressed satisfaction with the VR feeding simulation device, generally considering it effective and providing a pleasant experience. | The overall experience with the device was decent (452). The overall experience with the VR feeding simulation device was good (C471). The overall experience with the VR feeding simulation device was very satisfactory (C532). The overall experience with the VR feeding simulation device was acceptable, and the overall design was good (W119). |
|  | Novel | 5 | Patients affirmed the overall experience with the VR feeding simulation device, describing its design as novel and unique, which brought a fresh experience. | The overall experience with the VR feeding simulation device was good and rather novel (W135). The design of the device was good, very innovative, and something I hadn’t seen before (W119). The overall experience with the VR feeding simulation device was decent; it was quite novel and innovative (W81). |
|  | Enjoyable | 6 | Patients found the experience with the VR feeding simulation device to be interesting, fresh, and enjoyable, which enhanced the overall enjoyment of its use. | I feel that the overall experience with this device was good and fun (C474). It felt quite nice and interesting (W132). I have used this VR feeding simulation device for a few days, and the overall experience was quite good and enjoyable (W85). |
|  | Moderate | 4 | Some patients considered the overall experience with the device as average, feeling it was unremarkable and lacked special appeal, and noted that there is room for improvement. | I think the device is average, neither remarkable nor disappointing (C248). The overall experience with the VR feeding simulation device was ordinary, with no particular impressions (W130). The overall experience with this VR device was average, with some room for improvement (W175). |
|  | Immersive Experience | 13 | Patients were satisfied with the immersive experience of the VR feeding simulation device, feeling that it realistically recreated scenes of a Chinese restaurant, dessert shop, and fruit store, making them feel as if they were in a real dining environment where they could see and smell the food, and even experience a sense of presence. | I like the fruit scenes; the pastry shop scene was very realistic (C471). It made me feel as if I were actually eating, and I could enjoy various types of food (W155). The immersive experience with this VR device made me feel as if I were there (W153). I could see the appearance of the food and smell its scent (C478). The scent was nice, and I could distinguish what it smelled like (W81). |
|  | Easy to Use  Overall Experience | 5 | Patients reported that the device was easy to operate, convenient to use, could be used smoothly post-surgery, and was easy to master, allowing quick adaptation after an initial adjustment period. | Very user-friendly and convenient (C384). I was able to use it after surgery (C478). Practical and convenient, easy to promote (W152). I found it easy to learn how to use this device and quickly got the hang of it (W154). There was a bit of discomfort initially, but I adapted after using it for a few minutes and learned quickly (W177). |
|  | No Discomfort | 1 | Patients generally reported a positive experience using the device, finding the process enjoyable and experiencing no discomfort. | I feel that the overall experience with this device was good, fun, and without any discomfort (C474). |
|  | Particularly Enjoy Certain Scenes or Foods | 4 | Patients particularly enjoyed the Chinese food scenes presented in the device, especially Sichuan cuisine, such as Mapo tofu and hot pot, as the display of these foods stimulated their appetite. | I am older, and I liked the Chinese food scenes (C483). Especially the Sichuan cuisine, which looked very appetizing (W119). After using this device, seeing Sichuan dishes like Mapo tofu and hot pot stimulated my appetite significantly (W58). |
| Effect on Discomfort During Perioperative Fasting | Relieves Bloating | 7 | Some patients reported relief from abdominal bloating after using the device. | After using this device, my stomach no longer felt bloated (W152). It helped relieve my bloated feeling after surgery; my stomach didn’t feel as tight or painful after each use (W104). This VR feeding simulation device alleviated my postoperative bloating, and my stomach felt less distended after use (W81). |
|  | Promotes Intestinal Peristalsis | 4 | Patients reported feeling hunger and hearing stomach growling after using the device, indicating that it may stimulate the recovery of intestinal function. | After using the device, I felt hungry and could hear my stomach growling (C452). After using the device, my discomfort was relieved; I felt it stimulated the recovery of my intestinal function, as I could hear my stomach sounds (CW155). I feel that this device promotes bowel movement; I could hear my intestines gurgling (W175). |
|  | Facilitates Gas Expulsion | 4 | Some patients reported easier gas passage after using the device, believing it to be beneficial for postoperative gastrointestinal function. | After using the device, passing gas became easier (W154). This VR device helped my gastrointestinal function post-surgery; for instance, I could release trapped gas after using it (W175). |
|  | Improves Mood | 9 | Patients reported that using the device relieved discomfort from fasting, making them feel more at ease. | After using this VR device, the frustration of being unable to eat disappeared, and I felt more comfortable (C160). This VR feeding simulation device helped relieve the discomfort caused by fasting post-surgery, making me feel more at ease (C494). It had a significant effect on alleviating fasting-related discomfort, relieving my frustration from not being able to eat (C513). It had some effect in alleviating fasting-related discomfort, making me feel more pleasant (C481). This VR device provided psychological comfort when I couldn’t eat, easing some of the discomfort (W155). |
|  | Not Much Help | 7 | Some patients felt that the device did not significantly alleviate postoperative discomfort, describing the overall experience as average, with no special sensation. | I don’t particularly mind fasting due to surgery, so I feel the device did not provide much relief (C248). The effect was average; I didn’t feel much of a difference (C478). No significant help (W135). |
|  | Effect on Discomfort During Perioperative Fasting  Relieves Discomfort of Being Unable to Eat | 20 | Patients found the device helpful in alleviating the discomfort of craving food due to fasting during surgery. | I believe that using this device is helpful for dealing with the inability to eat post-surgery, especially when I feel like eating (W132). This VR feeding simulation device helped alleviate the discomfort caused by fasting due to surgery (C471). It had some effect in alleviating fasting-related discomfort, though not very pronounced (C483). This device helped with my desire to eat while fasting due to surgery, making me feel less uncomfortable afterward (W119). During surgery, I was prohibited from eating, and when I got hungry, the simulation and smell of food made me feel slightly better (W86). |
|  | Reduces Hunger | 3 | Patients believed the device helped alleviate hunger and nausea caused by fasting during surgery. | This device helped alleviate some of the hunger and nausea from fasting during surgery, providing some relief (W177). This VR feeding simulation device helped relieve hunger during preoperative and postoperative fasting, and the effect was good (C452). |
| Impact  on Appetite | Stimulates Appetite | 30 | Patients reported that the device effectively stimulated their appetite, helping them regain the desire to eat post-surgery. They noted that seeing virtual food and engaging in virtual dining significantly enhanced their appetite, even quickly rekindling their hunger. | It can stimulate my appetite, helping restore my desire to eat after surgery (W132). Seeing food and virtually dining boosted my appetite recovery (C474). This device quickly stimulated my appetite, making me crave food (C160). It was helpful; after using it, my appetite recovered, and I felt like eating (W104). After surgery, I already wanted to eat, and using this device made me want to eat even more; seeing the virtual food further stimulated my appetite (W175). |
|  | Slight or No Significant Impact on Appetite | 7 | Some patients felt that the device had little effect on their appetite, with feedback indicating that the change in appetite after use was minimal, with some even stating that they had no particular feeling. | Seeing the food didn’t help my appetite much (C248). After using this device, there was no obvious change in my appetite; I didn’t feel anything special (W81). While using this device, my appetite recovered a bit (W157). |
| Suggestions  for the Device | Add More Scenes | 1 | Patients expressed satisfaction with the design of the device and hoped for more virtual dining scenes to enrich their experience. | I am very satisfied with the design of this device; more scenes could be added (C494). |
|  | Expand Food Options | 9 | Some patients felt that the variety of food in the device was limited and suggested adding more dishes, including staple foods and Chinese cuisine, to enhance interest and effectiveness. | The variety of food in the VR feeding simulation scenes is limited; I hope more dishes can be added (C478, C532). The device is well-made; I hope more types of food, like staple foods and Chinese dishes, can be added, not just vegetables (C483). The device is good; it could have more functions and food types to keep me interested, leading to better therapeutic effects (W154). I hope the VR feeding simulation device includes a richer variety of foods, with more distinctive scents, especially local specialties (W58). |
|  | Scent Too Strong | 1 | Some patients reported that the scent of certain foods was too strong and pungent, suggesting that the odor be toned down. | Some food scents were a bit strong and pungent; they could be toned down slightly (C347). |
|  | Scent Too Faint | 2 | Other patients felt that the scent of some foods was too faint, lacking a sense of realism. | Some food scents were rather faint and felt flavorless (C248). Some food lacked authenticity, and certain scents weren’t strong enough (C478). |
|  | Excellent, No Suggestions | 18 | Some patients were very satisfied with the device, feeling that no improvements were necessary. | I have no suggestions; I think this device is quite good (W132). The device development was quite good; I have no suggestions (C348). The device design is good, and I currently have no suggestions (C452). |
|  | Enhance Realism | 3 | Some patients noted that certain foods lacked realism, expressing a desire for more realistic fiber textures in fruits and better visual representation of food. | Some foods lacked authenticity (C478). I am very satisfied; I hope the fruit textures could be made more realistic (C471). The food graphics could be made more realistic (C481). |
|  | Improve Background Music | 1 | Patients hoped for more pleasant background music in the scenes, as the current music made them feel drowsy. | I hope the background music in the scenes could be more pleasant, as the current music makes me feel sleepy (C478). |
|  | Desire for Broad Promotion and Adoption  Suggestions for the Device | 2 | Many patients found the device well-designed and hoped for its promotion in primary healthcare institutions to benefit more patients. | The device is well-made, and I hope it can be widely promoted (C521). The device is well-made, and I hope it can be made available to grassroots medical institutions, benefiting a broad range of patients (W152). |
|  | Simplify Operations | 3 | Some patients pointed out that the device was not convenient to use, especially when using the handle to pick up food during IV infusions, suggesting it be designed in a more user-friendly form. | The overall design of the device is quite good, but it’s inconvenient to use a handle to pick up food while my hand is receiving an IV drip; I hope it can feed me directly (W119). This could be designed more simply; currently, I need someone to teach me how to use it. I hope it can be designed to be ready-to-use (W175). The overall experience with the VR feeding simulation device was quite good, but using a handle to grab food is inconvenient post-surgery, as I need to operate it constantly with my hands (W87). |
|  | Optimize for Multiple Perspectives | 2 | Some patients hoped for an upward-looking perspective, allowing them to use the device while lying down. | The overall device design is quite good, but sometimes the viewing angle is inconvenient; I hope an upward viewing angle can be added so I can use it while lying down (W104). I hope an upward viewing angle can be added so that I can use it lying down, as I sometimes feel tired (W175). |
|  | Enhance Hardware | 1 | Some patients suggested that the headset could be made lighter for better comfort. | It would be better if the headset were lighter (W86). |
